# Neutropenia and agranulocytosis during treatment of schizophrenia with clozapine versus other antipsychotics: an observational study in Iceland

**DOI:** 10.1186/s12888-016-1167-0

**Published:** 2016-12-12

**Authors:** Oddur Ingimarsson, James H. MacCabe, Magnús Haraldsson, Halldóra Jónsdóttir, Engilbert Sigurdsson

**Affiliations:** 1 Faculty of Medicine, School of Health Sciences, University of Iceland, Reykjavík, Iceland; 2 Landspitali University Hospital, Mental Health Services, Hringbraut, 101 Reykjavik, Iceland; 3 Department of Psychosis Studies, Institute of Psychiatry, Psychology and Neuroscience, Kings College, London, UK; 4 National Psychosis Unit, Bethlem Royal Hospital, South London and Maudsley NHS Foundation Trust, London, UK

**Keywords:** Schizophrenia, Clozapine, Antipsychotics, Neutropenia, Agranulocytosis

## Abstract

**Background:**

Data on the haematological outcomes of patients who continue clozapine treatment following neutropenia are very rare as even mild neutropenia results in mandatory discontinuation of clozapine in most countries. However, in Iceland where clozapine monitoring is less stringent allows an observational study to be done on the risk of agranulocytosis and neutropenia during treatment with clozapine compared with other antipsychotics among patients with schizophrenia.

**Methods:**

The present study is a part of a wider ongoing longitudinal study of schizophrenia in Iceland. We identified 201 patients with schizophrenia treated with clozapine and 410 patients with schizophrenia who had never been on clozapine by searching the electronic health records of Landspitali, the National University Hospital. Neutrophil counts were searched in electronic databases to identify patients who developed neutropenia/agranulocytosis and the frequency of neutrophil measurements was examined as well.

**Results:**

The median number of days between neutrophil measurements during the first 18 weeks of clozapine treatment was 25 days but after the first 18 weeks on the drug the median became 124 days. Thirty four cases of neutropenia were identified during clozapine treatment with an average follow up time of 9.2 years. The majority, 24 individuals developed mild neutropenia (1500–1900 neutrophils/mm^3^). None of these progressed to agranulocytosis. The remaining 10 patients developed neutropenia in the range 500–1400 /mm^3^ of whom one developed agranulocytosis, three stopped clozapine use and 6 patients continued on clozapine for at least a year without developing agranulocytosis. Unexpectedly, schizophrenia patients on other antipsychotics had an equal risk of developing neutropenia as those on clozapine.

**Conclusions:**

Neutropenia is common both in patients with schizophrenia on clozapine treatment and in those never on clozapine. Therefore a large part of neutropenia during clozapine treatment is probably not caused by clozapine. These findings have implications in assessing the balance between the risk of progression from neutropenia to agranulocytosis against the morbidity resulting from the premature discontinuation of clozapine under the current monitoring regulations in the US and in most of Europe.

## Background

Around 20–30% of patients with schizophrenia prove to be treatment resistant, defined as failure to respond to two or more antipsychotics (one of which should be an atypical) when given an adequate dose for at least six to eight weeks [1, 2]. Clozapine has been demonstrated to be the drug of choice for these patients [3, 4].

Some physicians are reluctant to prescribe clozapine, probably because of the relatively high burden of adverse side effects, especially the rare but potentially life-threatening agranulocytosis [4]. Even though clozapine has a high burden of various adverse side effects we have reported that over 70% of patients that commence clozapine treatment in Iceland remain on it long term [5]. A pharmacogenetic test for agranulocytosis with adequate predictive validity is unlikely and would likely present ethical challenges [6, 7]. The risk of agranulocytosis is managed in most developed countries by mandatory blood monitoring for patients on clozapine [8]. In the UK patients taking clozapine must enrol in a clozapine monitoring service where it is obligatory to be monitored weekly for the first 18 weeks of treatment. During the next 34 weeks neutrophil monitoring is done every other week and then monthly after the first year of monitoring has passed [9]. The risk of agranulocytosis is estimated to be 0.68% but after the first year this risk drops by a factor of 10 [10, 11]. The mortality when agranulocytosis develops has been estimated to be 2.7–3.1% [12, 13]. Therefore the absolute mortality of patients on clozapine due to agranulocytosis is very low or around 0.02% or two out of ten thousand patients. Regular blood monitoring has been reported to be effective in reducing this risk [14] but it also places limitations on the use of clozapine in three ways: firstly, by excluding patients living in areas where clozapine monitoring systems are not in place, secondly by making physicians reluctant to prescribe it, thirdly by placing an additional burden on patients, and lastly by requiring that any patient whose neutrophil count drops below 1500/mm3 discontinues clozapine permanently [15]. There is growing evidence that clozapine treatment is associated with reduced mortality compared to treatment with other antipsychotics [16].

Monitoring systems are in place in the United States and in most European countries but the regulation of clozapine use varies substantially between countries [8]. Some countries, including Iceland, have taken a more flexible stance and not enforced such regulations for clozapine prescriptions in view of the fact that there is no other alternative drug available with similar efficacy for treatment resistant schizophrenia, as well as the practical difficulties of regular monitoring in remote areas.

People of certain ethnic groups, such as Yemenite Jews and 25–50% of black Africans, commonly have an intrinsic low neutrophil count in the range of 1.0 – 1.5 without any observed adverse clinical effects such as more frequent bacterial infections. These individuals are said to have benign ethnic neutropenia (BEN) [17]. According to the UK guidelines, clozapine treatment is stopped when neutrophil count falls below 1500/mm^3^. By following this recommendation it can be quite challenging to provide clozapine to these patients. This reality was acknowledged in the USA in the recent change of recommendations in October 2015 by the Food and Drug Administration (FDA) with a relaxation of the requirement to stop clozapine treatment when the neutrophil count falls below 1000/mm^3^ [18]. Prescribers can now continue to prescribe clozapine treatment for patients with an neutrophil count less than 1000/mm^3^ if the prescriber’s evaluation is that the benefits of clozapine therapy outweigh the risk of severe neutropenia [18].

The risks and benefits of changing or abolishing clozapine monitoring are difficult to quantify owing to a lack of evidence on the natural course of neutrophil counts in the absence of a monitoring programme.

### The aim of this study

To analyze the risk of neutropenia and further the progression to agranulocytosis in a sample of patients with schizophrenia in Iceland, where it is not mandatory to provide blood samples at certain intervals in order to get clozapine dispensed.

## Methods

### Study population

**Fig. 1:**
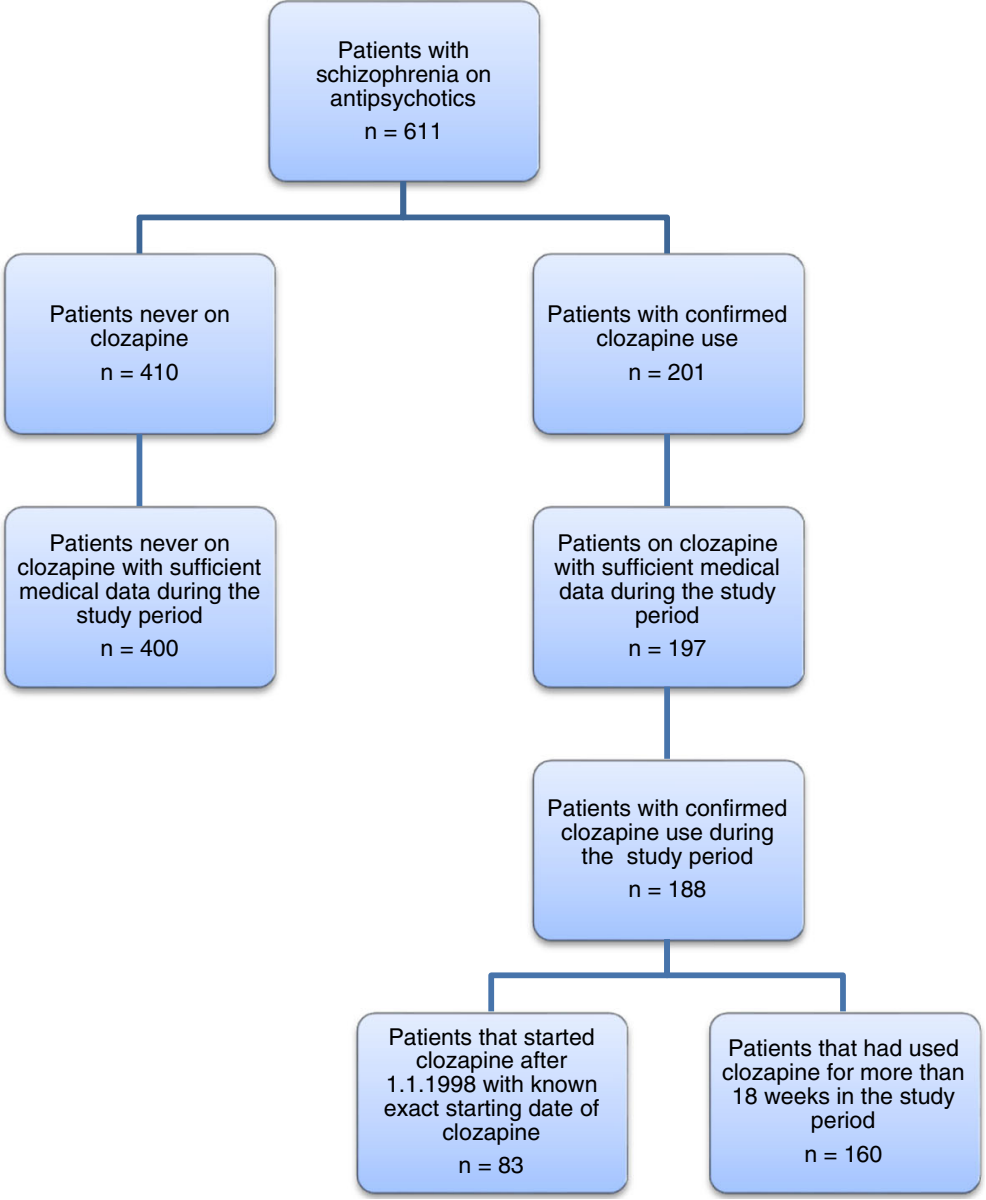
Description of study cohort in the study period of 1.1.1998 – 21.11.2014

The present study is a part of a wider ongoing longitudinal study of psychotic disorders in the Landspitali University Hospital (LUH) department of psychiatry focusing on patients with schizophrenia and bipolar disorder. Patients have been recruited to the study in several waves from 1986–2014. The majority of inpatients and outpatients at LUH with schizophrenia have been invited to take part in the study. Almost all the patients in study have been admitted to LUH. Most of the patients were recruited in 2000–2004. In this study we looked at patients in the LUH cohort who were alive on the 1.1.2003 and who had a confirmed diagnosis of schizophrenia according to the Research Diagnostic Criteria, assessed using the “Schedules for Affective Disorder and Schizophrenia-Lifetime version” (SADS-L) [19]. In total there were 611 patients who met the criteria (Fig. 1).

LUH introduced electronic health records (EHR) in 1998 but older health records are available on paper. Subsequently the proportion of medical, psychology and nursing data in the EHR has been steadily growing and currently includes almost all patient data in LUH.

LUH is the only tertiary hospital for mental health services in Iceland. LUH also provides secondary psychiatric services and psychiatric inpatient beds for over 90% of the population, the proportion being even higher for treatment resistant patients. Therefore the overwhelming majority of all Icelandic patients with schizophrenia who have ever taken clozapine have been in regular contact with mental health services or other services at LUH.

### Case identification

To identify patients that have used clozapine we used a keyword search in the EHR for the words “clozapin”, “closapin” and “Leponex”. The “e” at the end of clozapin was skipped because of possible spelling errors in the EMR but a keyword search of “clozapin” will find both “clozapin” and “Clozapine”. Leponex was the only brand name of clozapine in Iceland during the study period. All medical notes with the clozapine keywords were reviewed to confirm clozapine use. Wherever insufficient documentation of prior psychotic disorder and medication use was present in the EHR, the paper medical records were reviewed. We identified 201 patients with schizophrenia. The remaining 410 patients with schizophrenia who had never used clozapine but had been treated with other antipsychotics comprised the comparison group. Information on the first period of clozapine treatment for patients with schizophrenia was available for 195 patients out of 201.

### Frequency of blood tests

The frequency of blood measurements was analyzed for patients treated with clozapine from 1.1.1998 until 21.11 2014. The frequency of measurements was calculated by dividing the total time on clozapine treatment by the number of neutrophil measurements. The frequency of measurements during the first 18 weeks was analyzed separately from the subsequent time-period. The frequency analysis for neutrophil measurements only included patients who were using clozapine for the first time. Eighteen patients for whom the clozapine start date was not known to an exact date and four patients who did not live in the Reykjavik metropolitan area, where LUH is situated, were excluded from the blood monitoring analyzes for the first 18 weeks.

### Identification of neutropenia or agranulocytosis

We searched electronically all available results of blood measurements at LUH for neutrophil counts. The LUH database is linked to regional laboratory databases in Iceland using the Icelandic social security number (kennitala) which is a unique personal identifier for each Icelander. Among these is the database in the regional hospital in Akureyri where Iceland’s only other department of psychiatry is located in Iceland’s second largest hospital. This department provides mental health services for the remaining 10% of Iceland’s population. We also used a keyword search in the EHR to identify additional cases with the following keywords to find medical notes where neutropenia or agranulocytosis were mentioned; “Neutropaenia”, “neutropaenia”, “neutropenia”, “leucopaenia”, “leucopenia”, “kyrningafæ”, “hvítkornafæ”. The complete medical notes, electronic as well as on paper, were reviewed in order to confirm the diagnosis.

Neutrophil count in the range of 1500/mm^3^ – 1900/mm^3^ was defined as mild neutropenia. Icelandic guidelines recommend that if neutrophils counts are 1500/mm^3^-1900/mm^3^ then neutrophil monitoring should be increased to twice every week until the neutrophil count rises to 2000/mm^3^ or above, whereas if neutrophils fall below 1500/mm^3^ treatment should be stopped [15, 20]. Neutrophil count in the range 1000/mm^3^-1400/mm^3^ was defined as moderate neutropenia and neutrophil count in the range of 500/mm^3^-900/mm^3^ as severe neutropenia [20]. Agranulocytosis literally means the absence of circulating granulocytes but the term is used in clinical practice in psychiatry when the neutrophil count falls below 500/mm^3^ [20]. Neutrophil counts in Iceland are rounded to the nearest 100/mm^3^.

The STROBE guidelines for reporting the results of observational studies were followed. Statistical analyses were performed with STATA, version 13. A Cox proportional hazard model was used to assess factors associated with neutropenia. One patient was excluded from the Cox proportional hazards model because he used clozapine only for one day.

## Results

### Neutrophil monitoring

In Iceland the guidelines for neutrophil monitoring recommend weekly neutrophil counts for the first 18 weeks and monthly subsequently, but these guidelines are not enforced in a mandatory way [15]. Table 1 describes the frequency of neutrophil measurements after clozapine treatment was started. During the first 18 weeks of treatment a total of 16 out of 83 patients discontinued clozapine. Neutrophils were measured weekly or more often for only 12 out of 83 patients (14.4%). Neutrophil measurements were performed every 5 weeks or less for 22 patients (26.5%) and 7 patients had no neutrophil measurements done. The mean number of days between neutrophil measurements was 25 days (SD = 28) and the median number of days between blood tests was 18 days.

**Table 1:** Frequency of neutrophil measurements during clozapine treatment

Measurements	n	%
First 18 weeks (*n* = 83)		
Weekly	12	14.5
Every 1–2 weeks	22	26.5
Every 2–3 weeks	15	18.1
Every 3–4 weeks	6	7.2
Every 4–5 weeks	6	7.2
Less than every 5 weeks	22	26.5
After 18 weeks (*n* = 160)		
Monthly	6	3.8
Every 1–2 months	33	20.6
Every 2–3 months	25	15.0
Every 3–6 months	31	19.4
Every 6-12months	37	21.3
Less than every year	31	20.0

Only 6 out of 160 patients (3.8%) had at least monthly measurements of neutrophils carried out in line with guidelines. The median number of days between neutrophil measurements was 124 days. If the 7 patients who had no neutrophil measurements done are excluded the mean number of days between measurements is 229 (SD = 329).

### Progression from neutropenia to agranulocytosis

Table 2 shows how frequently neutropenia progressed to agranulocytosis in our sample. For mild to moderate neutropenia (1000-1900/mm3) no progression to agranulocytosis was observed for 32 cases. If we only include patients that stayed on clozapine for at least one year following mild to moderate neutropenia then none of 28 patients developed agranulocytosis. For patients with moderate to severe neutropenia (<1500/mm3) one patient out of 10 developed agranulocytosis. It is worth noting that only 2 out of 8 (25%) patients with moderate neutropenia discontinued clozapine compared with 28.8% discontinuation for all patients ever prescribed clozapine for treatment resistant schizophrenia in our sample.

**Table 2:** Progression of lowest neutrophil count to agranulocytosis

Lowest neutrophil count (excluding counts taken during episode of agranulocytosis)	Amber: Mild neutropenia 1500-1900/mm3	Red: Moderate neutropenia 1000-1400/mm3	Severe neutropenia 500-900/mm3
	*n* = 24	*n* = 8	*n* = 2
One year after lowest level of neutropenia			
Still on Clozapine	22	6	0
Clozapine discontinued	2	2	1
Developed agranulocytosis (and then discontinued)	0	0	1
Clozapine and agranulocytosis status unknown	0	0	0
Three years after lowest level of neutropenia			
Still on Clozapine	18	4	0
Clozapine discontinued	3	2	1
Developed agranulocytosis (and then discontinued)	0	0	1
Clozapine and agranulocytosis status unknown	3	2	0

### Neutropenia in patients with schizophrenia

Table 3 describes the results of neutrophil measurements with regard to low neutrophil count in the period 1.1.1998 – 21.11.2014. We found only one 56 year old patient on clozapine whose neutrophils dropped below 500/mm^3^, a count clinically defined as agranulocytosis. Clozapine was identified as the most likely contributing factor. Since this patient had been on clozapine for 28 years when the agranulocytosis developed it cannot be excluded that unknown age-related causes were at play [11, 12]. Neutrophil measurements in the moderate to severe range of 500-1400/mm^3^ were found in 9 patients in the clozapine group or 4.8% of the cohort, on all occasions these were 900/mm^3^ and above. Moderate to severe neutropenia in the never-on-clozapine group was found in 23 patients or 5.8% of the cohort, and all the neutrophil counts were 800/mm^3^ and above.

**Table 3:** Lowest neutrophil count in the clozapine group and never-on-clozapine group

	On clozapine		Never-on-clozapine	
Average age at follow up [Years]	51.2		58.1	
Average number of neutrophil measurements per patient	32.4		15.8	
Average time of follow up [Years]	9.2		13.8	
	*n* = 188	%	*n* = 400	%
No neutrophil measurementsperformed in time period	6	3.2	8	2.0
Never neutropenia, neutrophils 2000/mm3 or higher	148	78.7	335	83.8
Mild neutropenia, neutrophils 1500-1900/mm3	24	12.8	30	7.5
Moderate neutropenia, neutrophils 1000-1400/mm3	8	4.3	20	5.0
Severe neutropenia, neutrophils 500-900/mm3	1	0.5	3	0.8
Agranulocytosis, neutrophils 0-400/mm3	1	0.5	4	1.0

Patients in the never-on-clozapine group proved to have a similar risk of developing agranulocytosis in the long term since four patients out of 400 (1%) developed agranulocytosis during the same period of observation. However, two out of four did develop agranulocytosis while on cytotoxic treatment due to cancer. The third case was clinically thought to have been caused by interferon treatment for hepatitis C. A likely cause could not be identified in the fourth case but possible contributing factors were alcohol abuse, malnutrition and hepatitis C.

Table 4 shows Cox proportional hazards models exploring the factors associated with detecting various degrees of neutropenia. When the outcome under scrutiny was neutrophils in the range of 0-1900/mm^3^ then neutropenia was associated with more frequent testing, younger age and female sex. Being on clozapine was not significantly associated with risk for developing neutropenia.

**Table 4:** Cox proportional hazards model with possible factors associated with detecting moderate to severe neutropenia and agranulocytosis

	Hazard Ratio	Standard Error	Z-Score	95% CI	*P*-value
A. Neutrophils 0 - 1900/mm3					
Patient on clozapine	1.33	0.33	1.14	0.81-2.18	0.25
Sex (female)	1.82	0.40	2.76	1.19-2.80	0.01
Average age at risk	0.97	0.01	−3.75	0.96-0.99	<0.001
Measurements per year	1.14	0.02	6.32	1.09-1.18	<0.001
B. Neutrophils 0 – 1400/mm3 (equivalent to red result)					
Patient on clozapine	0.74	0.33	−0.68	0.31-1.76	0.50
Sex (female)	2.00	0.67	2.08	1.04-3.88	0.04
Average age at risk	0.98	0.01	−1.47	0.96-1.00	0.14
Measurements per year	1.17	0.04	4.66	1.10-1.26	<0.001
C. Neutrophils 1000 - 1900/mm3					
Patient on clozapine	1.44	0.37	1.39	0.86-2.40	0.18
Sex (female)	1.76	0.41	2.46	1.12-2.77	0.03
Average age at risk	0.97	0.01	−3.85	0.95-0.98	<0.001
Measurements per year	1.13	0.02	5.98	1.09-1.18	<0.001
D. Neutrophils 1500 – 1900/mm3 (equivalent to amber result)					
Patient on clozapine	1.86	0.58	1.99	1.00-3.43	0.05
Sex (female)	1.70	0.49	1.85	0.97-3.00	0.09
Average age at risk	0.96	0.01	−3.70	0.94-0.98	<0.001
Measurements per year	1.12	0.03	4.62	1.06-1.17	<0.001

A) *n* = 587, 89 cases. B) *n* = 587, 37 cases. C) *n* = 587, 80 cases. D) *n* = 587, 52 cases

## Discussion

Neutrophil monitoring in Iceland was far less frequent than guidelines recommend. Neutropenia was equally commonly observed in the clozapine group as in the group that had never used clozapine, although mild neutropenia was more common in patients on clozapine. Of the eight patients on clozapine who developed a moderate neutropenia (neutrophils 1000 – 1400/mm^3^), which would have resulted in mandatory cessation of clozapine in the UK, only two discontinued clozapine. The other six remained on clozapine a year later and none of these six suffered a further episode of neutropenia. Of two patients whose neutrophil count fell below 1000/mm^3^, one went on to develop agranulocytosis, having been of clozapine for 28 years.

The occurrence of mild (12.8%), moderate (4.3%) and severe neutropenia (0.5%) was fairly commonly identified in patients on clozapine despite the low frequency of measurements. Since more frequent measurements were significantly associated with neutropenia, it is likely that the occurrence of mild to moderate neutropenia would have been higher if the frequency of measurements had been in line with guidelines.

Moderate (5%) and severe neutropenia (0.8%) was more common in patients with schizophrenia who had never been on clozapine despite having neutrophil counts done around half as frequently as those on clozapine. Only mild neutropenia was found more frequently in clozapine-treated patients than in those never on clozapine.

It came as a surprise that being on clozapine was not associated with neutropenia in the range of 0-1400/mm^3^. For mild neutropenia in the range 1500-1900/mm^3^ being on clozapine was, on the other hand, significantly associated with almost doubling of the risk for neutropenia (hazard ratio of 1.86). Therefore, treatment with clozapine seemed to predict mild neutropenia, a phenomenon which is usually clinically insignificant but that can probably increase the likelihood of clozapine treatment being discontinued. Females had a higher risk of developing neutropenia with a hazard ratio ranging from 1.70-2.00, depending on the neutropenia range under observation. It has been reported that agranulocytosis during clozapine treatment is more common among females [12]. Neutropenia was also significantly more common with lower age which is in line with what has been shown in larger studies [11].

It can be concluded that most of the neutropenic episodes in our sample were transient and of no observable clinical significance. However, of those developing mild neutropenia (neutrophils 1500 – 1900/mm^3^), none subsequently developed agranulocytosis. One study has reported that the rate of neutropenia during clozapine treatment was 11.8% as compared to 17.6% for those on second generation antipsychotics [21]. These results concur with ours and indicate that mild to moderate neutropenia is not a good predictor for clozapine induced agranulocytosis.

In our never-on-clozapine group four cases of agranulocytosis were identified and in three cases a plausible explanation was identified but the main cause was speculative in the fourth case. If that patient had been on clozapine then the agranulocytosis would almost certainly have been attributed to clozapine treatment because no other probable causes were found. The risk of neutropenia in the clozapine group was similar to that in the never-on-clozapine group, indicating that in many if not most instances of neutropenia during clozapine treatment observed neutropenia may in fact not be caused by the clozapine treatment.

The progression from neutropenia to agranulocytosis was low in our sample. When we look at patients that had ever developed neutropenia in the range of 500–1900/mm^3^ and had used clozapine for at least a year after developing neutropenia or agranulocytosis, then only one patient out of 29 (3.4%) progressed to agranulocytosis. These results should though be interpreted with caution because the sample is small and we note that a 95% confidence interval for one event in 32 cases is 0 to 9.5%. The progression rate of neutropenia to agranulocytosis for patients with neutropenia in the more restrictive range of 500-1400/mm^3^ who continued to take clozapine for at least a year after developing neutropenia, was 1 out 7 (14.2%). Most guidelines recommend stopping clozapine treatment when neutrophils fall below 1500/mm^3^ so such data on patients who continue on clozapine following a “red result” is not often available. In a study by Hummer et al. 8 out of 68 patients were diagnosed with transient neutropenia that resolved without any change in treatment but the average neutrophil count was in the mild neutropenia range, 1780/mm^3^ [22].

The frequency of neutrophil measurements in Iceland during the first 18 weeks of treatment proved to be far lower than guidelines recommend, the median time between measurements being 18 days instead of weekly. The frequency of neutrophil measurements after the first 18 weeks were also far from what guidelines suggest, the median time between measurements being 124 days instead of monthly. The infrequent neutrophil measurements did, however, not lead to more frequent agranulocytosis than expected, and the only patient who developed agranulocytosis did so after being on clozapine for over 28 years and recovered fully. It is possible that the inclination to accept flexible neutrophil monitoring, accepting that many treatment resistant patients find it difficult, unpleasant or costly to turn up weekly for blood tests as outpatients, may have contributed to a higher proportion of patients staying on clozapine long term in Iceland than reported in some other studies [23, 24].

There are some limitations in our dataset. When analyzing the neutrophil monitoring data we had access to the blood test databases in LUH and the regional hospital in Akureyri. There are two private blood test facilities that we did not have access to and it is possible that some blood measurements were done there. However most patients with schizophrenia and nearly all those on clozapine are followed up from the LUH. Some neutrophil measurements in the database may not have been requested for the purpose of clozapine blood monitoring but for other medical reasons. Therefore the true neutrophil monitoring associated with clozapine may be a little bit more or less than we state in the results. The true prevalence of neutropenia might also be underestimated if it had been diagnosed in the private blood test facilities.

It is likely that many patients stop clozapine treatment because of low neutrophil measurements despite the risk of agranulocytosis being small in mild to moderate neutropenia. Our data suggest that the majority of patients who stop clozapine treatment after falling into the “red zone” (neutrophils < 1500/mm^3^) would probably not develop agranulocytosis were they to remain on treatment, particularly if their neutropenia is in the 1000-1500/mm^3^ range. Clozapine rechallenge should be assessed as an option in those patients if no other treatment has had the same observed clinical treatment effect as clozapine. The reduced mortality associated with clozapine treatment is substantial [16] and this is likely to outweigh the small risk of agranulocytosis in patients who continue clozapine treatment despite mild to moderate neutropenia. The risk of dying from agranulocytosis on clozapine has been estimated to be 0.02% [10–13]. We can contrast that risk to the risk of dying in a road traffic accident in Europe over a 40 year period which has been reported to be 0.37% (1- (1–9.3/100.000)^40^) [25]. Therefore, in Europe a patient on clozapine for 40 years has an 18.5 times (0.37%/0.02%) higher risk of dying in a road traffic accident than of dying from agranulocytosis.

There is a large population of schizophrenia patients who are currently suboptimally treated; having previously made a response to clozapine that was subsequently discontinued due to neutropenia. Particularly where the neutropenia was in the 1000–1500 range, consideration should be given for clozapine rechallenge in these cases, bearing in mind that 80% of patients can be successfully challenged with careful patient selection and monitoring [26]. There is a strong case for actively seeking out such patients for clozapine rechallenge, and this approach has been shown to be successful [27].

## Conclusions

Our data would support revisions to current European guidelines, as those called for by Cohen [28]. Specifically, our results suggest that consideration could be given to reducing the lower limit of the amber range, whereby clozapine can be continued with additional monitoring, from 1500 to 1000. This change would result in a much lower rate of clozapine discontinuation, and our findings suggest that the progression to agranulocytosis would remain a rare event. Such a limit has been introduced in the UK for patients with benign ethnic neutropenia [29] and is in line with the new FDA guidelines regarding clozapine monitoring [18].

## Abbreviations

CI: Confidence interval
EHR: Electronic health records
FDA: Food and drug administration
LUH: Landspitali University Hospital
NIHR: National Institute for Health Research
